# Supplementary figures and images for: A monoclonal antibody collection for C. difficile typing ?
Source: Gut Pathog. 2024 Jan 19;16:4. doi: 10.1186/s13099-023-00592-7 (PMC10797914; doi:10.1186/s13099-023-00592-7)

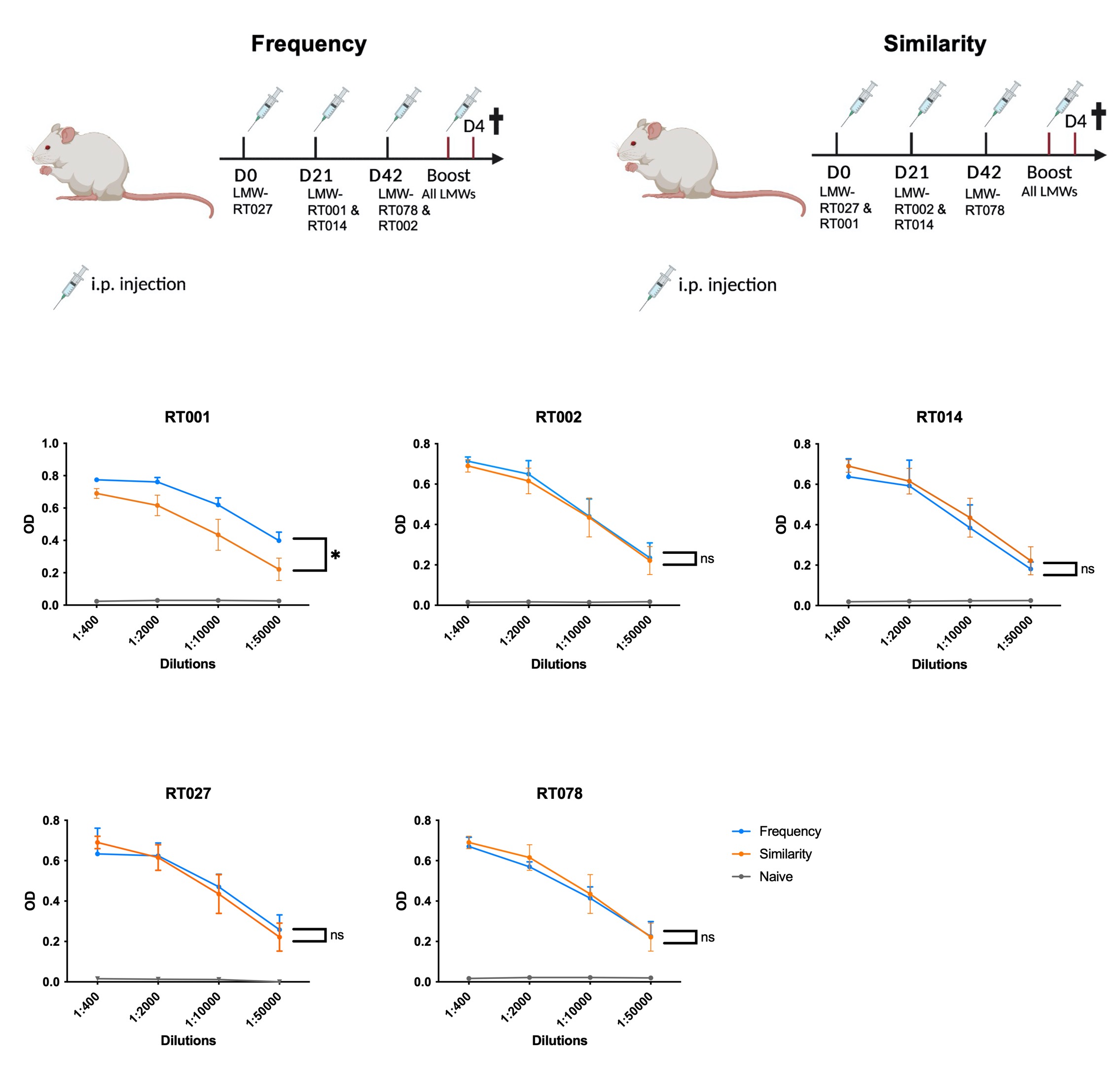

Supplement: Supplementary file 1 — Additional file 1: Figure S1. Comparison of two immunization protocols using recombinant LMWs. Mice were immunized following two different protocols termed “similarity” and “frequency”. a In the “Frequency” protocol, mice are immunized with LMWs in the order of their frequency in current CDI, and boosted with a mix of all five LMWs. In the “Similarity” protocol, mice are immunized with two highly similar LMW the same day, and boosted with a mix of all five LMWs. b Dose response of sera titers of immunized mice from the protocols depicted in (a) are measured by ELISA against the indicated LMW ribotype. Data are presented as mean values (±SD) for each group of mice (n = 5). ns: non-significant; *: p<0.05. Black curves represent sera from naive mice prior immunization. [file 13099_2023_592_MOESM1_ESM.jpg]
